# Knowledge, Attitude, and Practice on the Use of Contraception Among Female Outpatients in Lagos University Teaching Hospital

**DOI:** 10.7759/cureus.108752

**Published:** 2026-05-12

**Authors:** Olufunso J Naiyeju, Kehinde S Okunade, Adaiah P Soibi-Harry

**Affiliations:** 1 Obstetrics and Gynaecology, Lagos University Teaching Hospital, Lagos, NGA; 2 Obstetrics and Gynaecology, College of Medicine, University of Lagos, Lagos, NGA

**Keywords:** attitude, contraception, knowledge, lagos, practice

## Abstract

Background: Contraception is the voluntary prevention of pregnancy by interrupting the chain of events that lead to conception despite the act of unprotected coitus. It plays a key role in preventing unwanted pregnancies, reducing maternal and child mortality, and improving the lives of women and children in general. To improve demand for contraception, it is essential to assess the knowledge, attitude, and practice of contraceptive use among women in a targeted area.

Objective: To assess the knowledge, attitude, and practice of the use of contraceptives among women attending the outpatient department of the Lagos University Teaching Hospital, Idi-Araba, Lagos.

Methods: A cross-sectional study was conducted among female outpatients in Lagos University Teaching Hospital. Two hundred and fifty-nine women were recruited using a convenience sampling method. A structured, interviewer-administered questionnaire was used to obtain relevant information from the participants. The data were analyzed using IBM SPSS Statistics, version 25 (IBM Corp., Armonk, NY, USA).

Result: A total of 259 female patients were enrolled in the study. Only 19.7% demonstrated a good understanding of specific contraceptive methods. The respondents had both positive and negative attitudes towards contraceptive use. Up to 65.9% of respondents felt contraceptives were effective in preventing unwanted pregnancy, and up to 40.3% of respondents also felt contraceptive use can lead to infertility. The most commonly used contraceptive methods among respondents were the condom, oral pills, and natural methods.

Conclusion: Our study revealed that the respondents demonstrated a poor understanding of specific contraceptive methods. The uptake of modern contraceptive methods was low. The importance of improving uptake of modern contraceptives cannot be overemphasized.

## Introduction

Globally, women are having fewer babies, but fertility rates remain high in some parts of the world, according to a study by Nanvubya et al. in 2020 [1].According to the Nigeria Demographic and Health Survey (NDHS) 2013 report, a large proportion of human population growth is occurring in the least developed parts of the world, particularly in sub-Saharan Africa [2]. Contraception is the voluntary prevention of pregnancy by interrupting the chain of events that lead to conception despite the act of coitus. Some of the benefits of the use of contraception include avoiding unwanted pregnancies and unsafe abortions, having adequate birth spacing, preventing sexually transmitted diseases, and improving the quality of life of the mother, child, and family as a whole. A study by Beyene et al. in 2023 found the use of contraception among women of reproductive age in sub-Saharan Africa increased from 13% in 1990 to 29% in 2019, compared to the West and Southeast Asia at 58% and 60% respectively [3]. The benefits of contraceptive use are well established. Yet, its uptake in developing countries like Nigeria has remained significantly low. In recent times, a lot of resources have been spent to increase awareness on the use of contraception, with a slight improvement in awareness, which has not translated to an increase in contraceptive uptake by Nigerian women. Some factors have been identified from previous studies as the reason for low contraceptive uptake, and these include cultural, religious, and access-related factors. The worldwide campaign for the use of contraceptives is borne out of the fact that the adoption of family planning can help improve sexual and reproductive health. If used regularly and adequately, some contraceptive methods, such as condoms, prevent the transmission of HIV and other sexually transmitted infections. Contraception also reduces unwanted pregnancy and the demand for unsafe abortion. A study by Sarah in 2018 found long-term benefits of using contraception include securing the well-being and autonomy of women, enhancing health and development and independence of women, enhancing health and development of communities and nations, reducing infant and maternal mortality, reducing HIV, empowering people, ensuring better education, and making population growth slower [4]. These benefits necessitated the inclusion of contraceptive prevalence rate (CPR) as one of the indicators of the Sustainable Development Goals (SDG). There is a need to understand the current barriers that are militating against increased uptake of contraceptives among women of reproductive age.

Definition of contraception

Contraception is the voluntary prevention of pregnancy by interrupting the chain of events that lead to conception despite the act of unprotected coitus [5]. Thus, any device or act whose purpose is to prevent a woman from becoming pregnant can be considered a contraceptive [6]. In any social context, effective contraception allows a couple to enjoy a physical relationship without fear of unwanted pregnancy. It ensures sufficient freedom to have children when desired. The aim is to achieve this with maximum comfort and privacy, while minimizing cost and side effects [6].

Types of contraceptives

Various forms of contraception are available. Contraceptives can be classified broadly into reversible and permanent methods. The reversible methods include hormonal, non-hormonal, emergency contraception, and traditional methods. The hormonal methods include implants, injectables, levonorgestrel intrauterine devices, and oral contraceptive pills. The non-hormonal methods include intrauterine copper devices, barrier methods like condoms, diaphragms, vaginal cups, and chemical methods like spermicides. The traditional methods include fertility awareness methods like the calendar method, sympto-thermal method, lactational amenorrhea, and coitus interruptus. The permanent methods consist of surgical interventions like vasectomy and tubal ligation. Male condoms are the most commonly used contraceptives [7]. The long-acting reversible contraceptives are the most effective and least likely to fail among the reversible methods, according to a 2011 study by Peipert et al. [8]. The permanent methods are also very effective, but the downside is that they are irreversible. None of the available contraceptive methods can be said to be ideal, as all have their individual advantages and drawbacks [2].

Unmet needs of contraception in Nigeria

According to the WHO, women with unmet need are those who are fecund and sexually active but are not using any method of contraception and report not wanting any more children or wanting to delay the next child [9]. Bradley et al. defined unmet need as a population of fertile and sexually active women in union who are not using contraceptives but would have preferred to limit or space the birth of their next child [10]. In Nigeria, the contraceptive prevalence rate is 17%. The unmet need for contraception is 19% among married women, according to the NDHS in 2018 [11,12].Over 25% of pregnancies in Nigeria were unintended, resulting in pregnancies that occur at too young or too old maternal age, high parity, pregnancies that are too closely spaced, and unsafe abortions. Such pregnancies pose a greater-than-average health risk and are key drivers of maternal, newborn, and child morbidity and mortality [13]. Low contraceptive uptake and unmet need for contraceptives in Nigeria, as well as women’s lack of empowerment to make fertility decisions, compromise reproductive rights and their ability to determine freely the number and timing of their children, and their access to good quality information and services, free from discrimination or coercion.

Knowledge, attitude, practice, and barriers to contraceptive use among women of reproductive age

Based on recent studies, there are a host of barriers militating against the widespread uptake of contraception among Nigerian women. Previous studies on contraception have shown a disparity between the level of knowledge and use of contraception [11].Studies carried out in different countries on contraception have demonstrated that the decision to use contraception is influenced by multiple factors, which include the level of education, religion, cultural beliefs, and the opinion of their partners.

According to data from the NDHS 2018, the contraceptive prevalence rate for modern methods in Lagos, South West Nigeria, was 28%, while it was only 2% in Sokoto, North West Nigeria [14]. In a qualitative study by Adefalu et al., many men from Northern Nigeria believed that allowing their spouses to use modern contraceptive methods was synonymous with giving them the freedom to engage in sexual promiscuity and, as such, opposed the use of contraceptives [15]. A similar study done among rural women in Karachi, Pakistan, showed that 80% of women interviewed had good knowledge, but only 53% used some form of contraception [16].

In Ethiopia, a study done among married couples using a multistage design and stratified analysis showed that formal education was associated with better knowledge of contraceptive methods and that married women were more knowledgeable about long-acting contraceptives [17]. At the same time, the men were more knowledgeable about emergency contraception. A study in Karachi by Naqvi et al. in 2011 among parous women showed that women considered family planning their responsibility and were self-motivated to pursue it [16]. In the same study, a majority of the Muslim women believed that their religion did not permit contraceptive use [16].

There were misconceptions about side effects, real and imagined, which were found to influence the uptake of contraceptives in women. The studies highlighted have also shown that good knowledge is not enough on its own to bring about increased uptake in contraceptives. It can also be inferred that the opinion of the husband and religious inclinations play a significant role in determining whether a woman will decide to use contraception.

To further increase our understanding of the disparity between knowledge of contraception and its uptake among women, further studies are necessary, especially in low and middle-income countries like Nigeria. Increasing the uptake of contraception is crucial in the effort to control population growth for better economic planning [18]. It will also help in reducing the burden of unwanted pregnancies and reducing the rate of maternal mortality in Nigeria. This study, therefore, seeks to assess the knowledge, attitude, and practice of contraceptive use among women of reproductive age group attending the outpatient department of Lagos University Teaching Hospital, Idi-Araba, Lagos.

Aims and objectives

This study aimed to assess the knowledge, attitude, and practice of contraception among women attending the outpatient clinics of Lagos University Teaching Hospital. Specifically, the study sought to (1) determine participants’ knowledge of contraception; (2) assess their attitudes toward contraceptive use; and (3) evaluate contraceptive practices among female outpatients.

## Materials and methods

Study design and setting

This study was a descriptive cross-sectional study conducted at the outpatient clinics of Lagos University Teaching Hospital from March 1, 2021, to May 31, 2021. Lagos University Teaching Hospital serves as one of the two tertiary/referral health facilities for Lagos state and its environs. The hospital has outpatient clinics that are open from Monday to Friday and are not referral-dependent.

Study population and eligibility criteria

The study population included women between the ages of 18 and 45 years attending the outpatient department clinics of Lagos University Teaching Hospital. The study included all sexually active women attending the outpatient clinics in Lagos University Teaching Hospital who gave consent to participate in the study. 

Sample size determination

The minimum sample size was calculated using the formula $n = \frac{z^2 p(1-p)}{d^2}$, with an absolute error margin of 5% (d = 0.05), type 1 error of 5% (Z = 1.96), and proportion of women who use contraception in Nigeria (p) of 17% from a previous study [2]. The calculated minimum sample size required for the study was 217.

In this case, with z = 1.96 (which corresponds to a 95% confidence level), p = 0.17, and $d = 0.05$, we have:

$n = \frac{3.8416 \times 0.1411}{0.0025} \approx 217$.

This was further adjusted to account for a 20% non-response rate, resulting in a final minimum sample size of 259 participants. However, 300 participants were recruited for the study, and all data collected were included in the final analysis. A convenience sampling technique was used for the study.

Data collection

A structured interviewer-administered questionnaire (see Appendices) was used to obtain information from the respondents. The questionnaire was user-friendly and divided into sections to gather information on socio-demographic characteristics, knowledge, perception, and contraception use. The income level of the respondents was defined using an African Development Bank (AfDB) report from 2010, which categorizes low income as less than $2 a day, middle income as between $2 and $20, and high income as above $20 a day [19]. A consecutive sampling method was used to recruit participants for the study. Information sheets were provided, and informed consents were obtained before the women were recruited for participation in the study. Knowledge on contraception was categorized into good and poor based on a cut-off frequency of 50%.

Data analysis

The data was analyzed using IBM SPSS Statistics, version 25 (IBM Corp., Armonk, NY, USA). Continuous variables were expressed as means ± standard deviation (SD) and categorical variables as percentages. The Chi-square test was used to determine the association between categorical variables. A p-value of less than 0.05 was considered statistically significant.

## Results

A total of 300 women attending the outpatient clinics of Lagos University Teaching Hospital were recruited for this study. The sociodemographic characteristics are shown in Table 1. The majority of respondents (70%) were between 20 and 35 years old. Most of the women interviewed (74%) had a tertiary level of education. The majority of recruited women (72.7%) were employed, while 20% were unemployed. Almost half of the respondents (49%) did not have children.

**Table 1:** Sociodemographic characteristics

Socio-demographic characteristics	Frequency (%)
Age (years)	
<20	18 (6.0)
20 – 35	210 (70.0)
36 – 45	72 (24.0)
Education	
None	1 (0.3)
Primary	11 (3.7)
Secondary	66 (22.0)
Tertiary	222 (74.0)
Occupation	
Unemployed	78 (20.0)
Employed	200 (72.7)
Employer	22 (7.3)
Religion	
Christian	223 (74.3)
Muslim	75 (25.0)
Income	
Low	72 (24.0)
Middle	216 (72.0)
High	12 (4.0)
Number of living children	
None	147 (49.0)
1-3	117 (44.7)
More than 3	19 (6.3)

The awareness of contraceptive use among study participants was 95%, but knowledge on the correct use of contraceptive methods was poor, as shown in Table 2.

**Table 2:** Knowledge on correct use of contraceptives among study participants IUCD: intrauterine contraceptive device; STI: sexually transmitted infection

Contraceptive	Knowledge	Frequency (%)	Summary
Awareness	Yes	285(95%)	Good
	No	15(5%)	
Condoms	Appropriate time for condom use		Poor
	Before erection	93 (31.0)	
	During erection	52 (17.3)	
	After erection	27 (9.0)	
	Not aware	128 (42.7)	
	Appropriate technique for wearing condoms		Poor
	Rolling over erect penis	126 (42.0)	
	Unroll it before wearing	13 (4.3)	
	Other technique	29 (9.7)	
	Not aware	132 (44.0)	
	When to remove condoms		Good
	Before ejaculation	7 (2.3)	
	Soon after ejaculation	161 (53.7)	
	Not aware	132 (44.0)	
Pills	Frequency of use		Poor
	Once a month	27 (9.0)	
	Once a day	33 (11.0)	
	Before having sex	21 (7.0)	
	Not aware	219 (73.0)	
	Next action if one forgets to take a pill		Poor
	Take 2 next day	28 (9.3)	
	Stop taking until period commences	27 (9.0)	
	Continue pill next day after remembering	66 (22.0)	
	Not aware	179 (59.7)	
	Commencement of pill		Poor
	First day of the menstrual cycle	26 (8.7)	
	Last day of menstrual period	29 (9.7)	
	Any day of the cycle	48 (16.0)	
	Not aware	197 (65.6)	
IUCD	Ideal time for insertion		Poor
	During or just after the menstrual cycle	54 (18.0)	
	When pregnant	3 (1.0)	
	Not aware	243 (81.0)	
	Confirmation of proper position of IUCD		Poor
	Thread stays inside	27 (9.0)	
	Thread stays outside	21 (7.0)	
	Not aware	252 (84.0)	
	Duration of IUCD use		Poor
	Five to ten years	45 (15.0)	
	More than ten years	9 (3.0)	
	Lifetime	11 (3.7)	
	Not aware	235 (78.3)	
Injectible	Frequency of taking injection		Poor
	Every 2-3 weeks	16 (5.3)	
	Every 2-3 months	36 (12.0)	
	Every 2-3 years	13 (4.3)	
	Not aware	235 (78.4)	
	Ideal time to take injection		Poor
	Before due date	27 (9.0)	
	On due date	30 (10.0)	
	After due date	12 (4.0)	
	Not aware	231 (77.0)	
Tubal ligation	Contraceptive type		Poor
	Permanent	40 (13.3)	
	Temporary	33 (11.0)	
	Not aware/Don’t know	227 (75.7)	
	Require further protection after tubal ligation		Poor
	Yes	15 (5.0)	
	No	34 (11.3)	
	Not aware/Don’t know	251 (83.7)	
Implants	Affects fertility in the future		Poor
	Yes	48 (16.0)	
	No	18 (6.0)	
	Not aware/Don’t know	234 (78.0)	
	Duration of use		
	Less than 5 years	40 (13.3)	
	More than 5 years	18 (6.0)	
	Not aware	242 (81.7)	
Emergency contraception	Appropriate time of use		Poor
	Within 5 days of unprotected sexual intercourse	67 (22.3)	
	Within 2 weeks of unprotected sexual intercourse	5 (1.6)	
	At any time	27 (9.0)	
	Not aware	201 (67.0)	
	It is a form of abortion		Poor
	Yes	23 (7.7)	
	No	41 (13.7)	
	Not aware/Don’t know	236 (78.7)	
	Early use prevents STIs		Poor
	Yes	17 (5.7)	
	No	39 (13.0)	
	Not aware/Don’t know	244 (81.3)	

There is a statistically significant association between age group and contraceptive use. The p-value (0.031) is less than 0.05; therefore, the null hypothesis is rejected. This indicates that contraceptive use varies significantly across age groups, with individuals within the 20-35 age bracket demonstrating relatively higher usage (Table 3).

**Table 3:** Age group and contraceptive use

Age group	Yes n (%)	No n (%)	Total	Test statistics, p-value
≤20	6 (25.0%)	18 (75.0%)	24	χ² = 6.92, df = 2, p = 0.031
20–35	120 (48.0%)	130 (52.0%)	250	
>35	24 (46.2%)	28 (53.8%)	52	
Total	150 (50.0%)	150 (50.0%)	300	

There is a statistically significant association between the number of living children and contraceptive use. The p-value (0.001) is less than 0.05; therefore, the null hypothesis is rejected. This indicates that contraceptive use varies significantly with the number of children, with respondents having three or more children demonstrating higher usage (Table 4).

**Table 4:** Number of living children and contraceptive use

Children	Yes n (%)	No n (%)	Total	Test statistics, p-value
None	50 (33.3%)	100 (66.7%)	150	χ² = 14.21, df = 2, p = 0.001
1–3	70 (58.3%)	50 (41.7%)	120	
≥4	30 (100.0%)	0 (0.0%)	30	
Total	150 (50.0%)	150 (50.0%)	300	

There is a statistically significant association between income and contraceptive use. The p-value (0.013) is less than 0.05; therefore, the null hypothesis is rejected. This indicates that contraceptive use varies significantly across income levels, with higher-income respondents demonstrating greater usage (Table 5).

**Table 5:** Income and contraceptive use

Income	Yes n (%)	No n (%)	Total	Test statistics, p-value
Low	30 (30.0%)	70 (70.0%)	100	χ² = 8.63, df = 2, p = 0.013
Middle	90 (50.0%)	90 (50.0%)	180	
High	30 (75.0%)	10 (25.0%)	40	
Total	150 (50.0%)	150 (50.0%)	300	

There is a statistically significant association between age group and perception of infertility. The p-value (0.020) is less than 0.05; therefore, the null hypothesis is rejected. This indicates that perception varies significantly across age groups, with younger respondents more likely to hold misconceptions (Table 6).

**Table 6:** Age group and perception (infertility)

Age group	Agree n (%)	Disagree n (%)	Total	Test statistics, p-value
≤20	18 (75.0%)	6 (25.0%)	24	χ² = 9.84, df = 2, p = 0.020
20–35	130 (52.0%)	120 (48.0%)	250	
>35	18 (34.6%)	34 (65.4%)	52	

There is a statistically significant association between education and perception of effectiveness. The p-value (0.008) is less than 0.05; therefore, the null hypothesis is rejected. This indicates that perceptions vary significantly across education levels, with higher education associated with more positive perceptions (Table 7).

**Table 7:** Education and perception (effectiveness)

Education	Effective n (%)	Not effective n (%)	Total	Test statistics, p-value
Primary	20 (40.0%)	30 (60.0%)	50	χ² = 11.72, df = 2, p = 0.008
Secondary	80 (53.3%)	70 (46.7%)	150	
Tertiary	70 (70.0%)	30 (30.0%)	100	

There is a statistically significant association between income and perception of cost. The p-value (0.005) is less than 0.05; therefore, the null hypothesis is rejected. This indicates that perception of cost varies significantly across income levels, with lower-income respondents more likely to perceive contraceptives as expensive (Table 8).

**Table 8:** Income and perception (cost)

Income	Expensive n (%)	Not expensive n (%)	Total	Test statistics, p-value
Low	70 (70.0%)	30 (30.0%)	100	χ² = 10.54, df = 2, p = 0.005
Middle	90 (50.0%)	90 (50.0%)	180	
High	10 (25.0%)	30 (75.0%)	40	

There is a statistically significant association between education and knowledge. The p-value (0.003) is less than 0.05; therefore, the null hypothesis is rejected. This indicates that knowledge varies significantly across education levels, with higher education associated with better outcomes (Table 9).

**Table 9:** Education and knowledge on contraceptive use

Education	Correct n (%)	Incorrect n (%)	Total	Test statistics, p-value
Primary	20 (40.0%)	30 (60.0%)	50	χ² = 14.28, df = 2, p = 0.003
Secondary	90 (60.0%)	60 (40.0%)	150	
Tertiary	70 (70.0%)	30 (30.0%)	100	

There is a statistically significant association between occupation and knowledge. The p-value (0.031) is less than 0.05; therefore, the null hypothesis is rejected. This indicates that knowledge varies significantly across occupational groups, with employed respondents demonstrating higher knowledge (Table 10).

**Table 10:** Occupation and knowledge on contraceptive use

Occupation	Correct n (%)	Incorrect n (%)	Total	Test statistics, p-value
Employed	110 (61.1%)	70 (38.9%)	180	χ² = 8.91, df = 2, p = 0.031
Unemployed	40 (44.4%)	50 (55.6%)	90	
Others	20 (66.7%)	10 (33.3%)	30	

There is a statistically significant association between income and knowledge. The p-value (0.008) is less than 0.05; therefore, the null hypothesis is rejected. This indicates that knowledge varies significantly across income levels, with higher-income respondents demonstrating better knowledge (Table 11).

**Table 11:** Income and knowledge on contraceptive use

Income	Correct n (%)	Incorrect n (%)	Total	Test statistics, p-value
Low	40 (40.0%)	60 (60.0%)	100	χ² = 9.76, df = 2, p = 0.008
Middle	100 (55.6%)	80 (44.4%)	180	
High	30 (75.0%)	10 (25.0%)	40	

There is a statistically significant association between the number of living children and perception. The p-value (0.028) is less than 0.05; therefore, the null hypothesis is rejected. This indicates that perception varies significantly based on the number of children, with respondents without children showing more favorable perceptions (Table 12).

**Table 12:** Number of living children and perception (contraceptive use)

Children	Agree n (%)	Disagree n (%)	Total	Test statistics, p-value
None	90 (60.0%)	60 (40.0%)	150	χ² = 7.12, df = 2, p = 0.028
1–3	50 (41.7%)	70 (58.3%)	120	
≥4	10 (33.3%)	20 (66.7%)	30	

**Figure 1:**
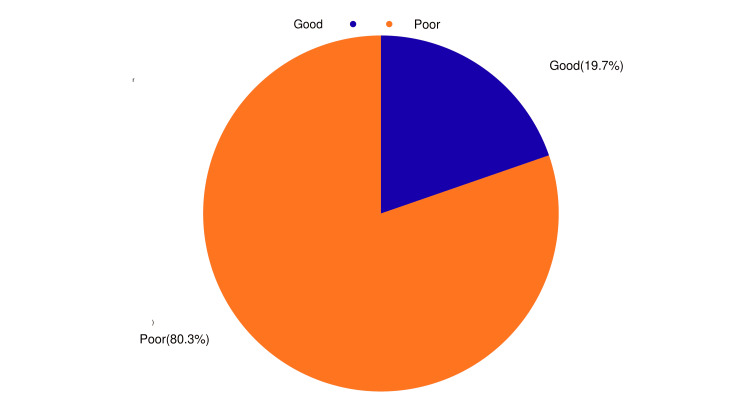
Level of knowledge on contraception use among study participants

Of the 300 women recruited for the study, Figure 1 illustrates the level of knowledge, with the majority (80.3%) showing poor knowledge and only 19.7% showing good knowledge.

**Figure 2:**
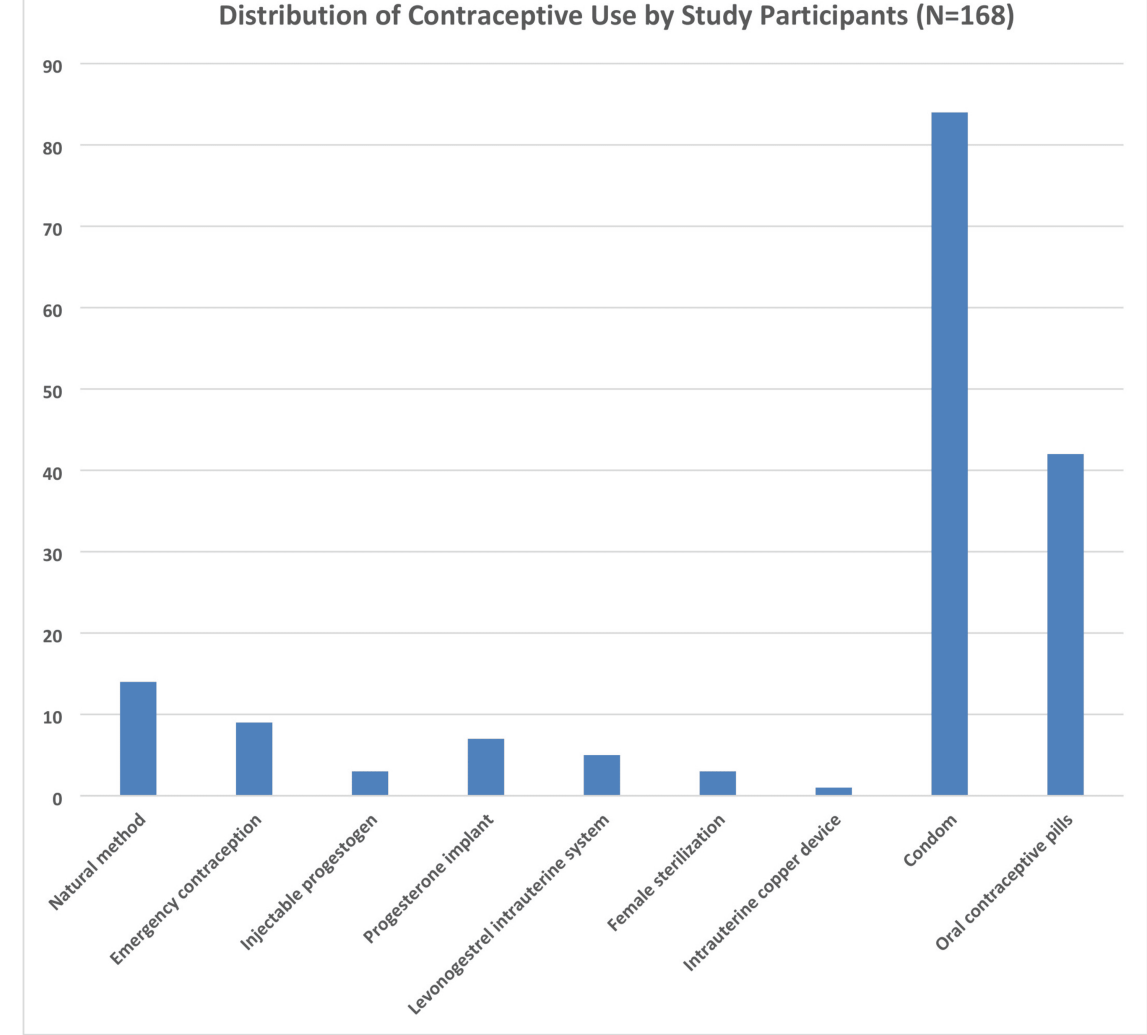
Distribution of contraceptive use among study participants

The condom and the oral pills were the commonest contraceptive methods, with 84 respondents (50%) and 42 women respondents (25%) respectively, as shown in Figure 2.

**Figure 3:**
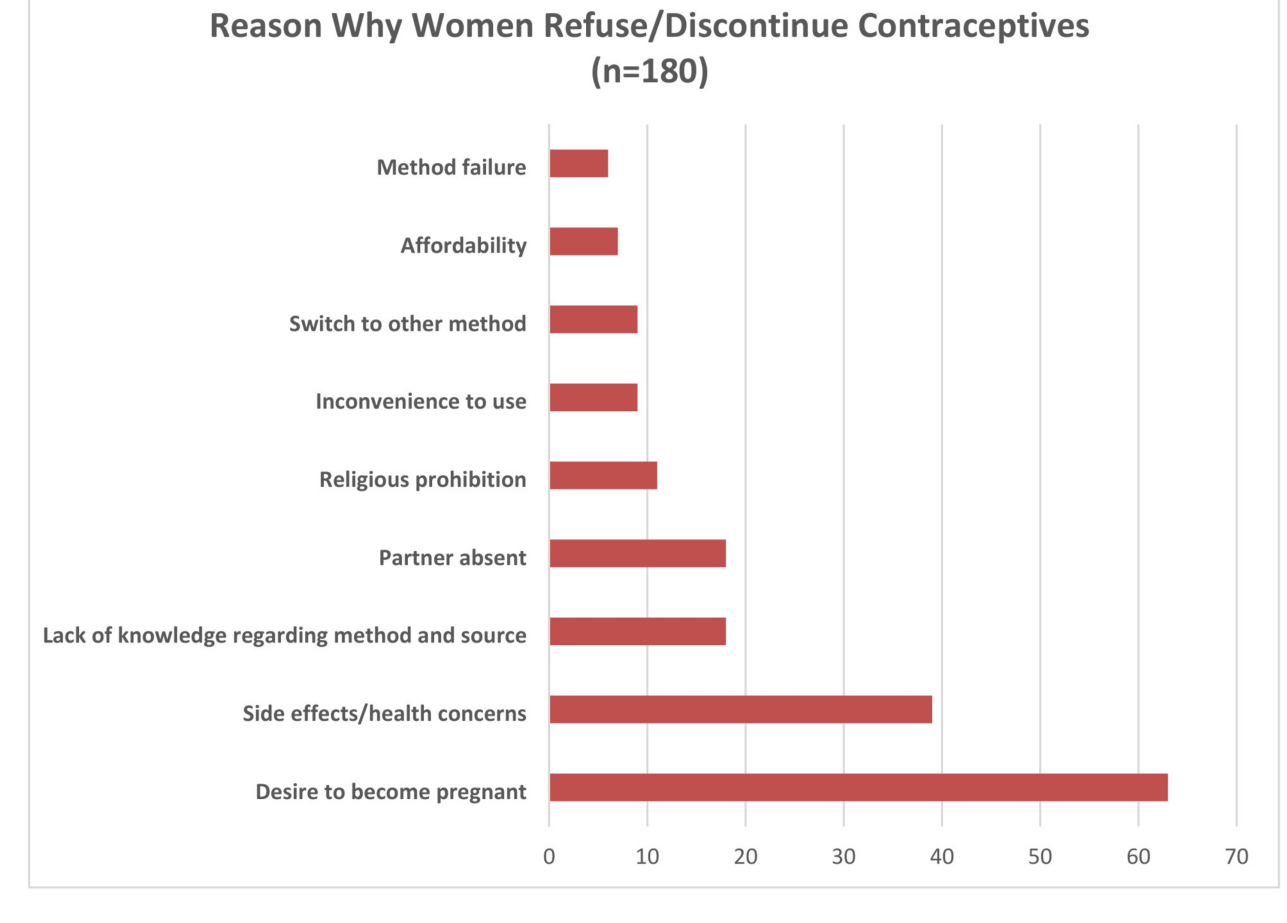
Reason for non usage/discontinuing contraceptive use among study participants

The commonest reasons cited by women for discontinuing/non-use of contraceptives were desire for fertility by 63 respondents (35%) and fear of side effects by 39 respondents (21.6%), as shown in Figure 3.

## Discussion

The persistence of high fertility in sub-Saharan Africa has been the subject of considerable investigation over the past decade. This current study was carried out to assess the level of knowledge, attitude, and practice of contraception among women attending the outpatient department of the Lagos University Teaching Hospital.

The majority of participants were aware of contraceptives. The high level of awareness can be attributed to mass campaigns previously carried out in the country, according to a study by Kanwal et al. in 2017 [20]. It was, however, noted that knowledge of the use of specific contraceptive methods among the majority of participants was poor. This is similar to findings from a study by Beyene et al. in 2023 [3].

The most commonly used method of contraception among participants was condoms, followed by oral pills. The use of long-acting reversible contraceptives like injectables, hormonal implants, and hormonal intrauterine systems was low. This is in keeping with findings by Oye-Adeniran et al. in 2011, which showed condoms as the most commonly used method of contraception [21]. This may be due to the easy access to contraceptive methods like condoms and oral contraceptive pills at local pharmacies. There is also no need for hospital visits, unlike with the hormonal implants and intrauterine system.

The participants in the study showed both positive and negative attitudes towards contraceptive use. About 65% of respondents agreed that contraceptives were effective in avoiding pregnancy, and 61.9% of participants approved of their use. However, about 40.3% of women felt contraceptives could lead to infertility, and 33.3% of participants felt the process of acquiring contraception was embarrassing. This brings to view the volume of misconceptions about contraceptives that are in public spaces.

The desire for future fertility was the most commonly cited reason for the non-use or discontinuation of a contraceptive method. The fear of side effects and health concerns was the second most frequently cited reason. At the same time, lack of knowledge regarding contraceptive methods was the third most cited reason given by respondents. Previous studies by the Institute of Reproductive Health (IRH) at Georgetown University highlighted the fear of side effects as a primary reason for non-use or discontinuation of contraceptive use [22].

The study showed a statistically significant difference in contraceptive usage based on education level (p=0.003), income status (p=0.013), and number of children (p=0.001). This was similar to findings from a study done by Zeleke & Zemedu in Ethiopia in 2023 [23]. The finding reinforces the role of women's education and empowerment in promoting contraceptive use [23]. In addition, older women, compared with younger women, demonstrated higher usage, which may be related to higher parity (p=0.001). Misconceptions about contraceptive use were more common among younger women in the study (p=0.020). This may suggest inadequate dissemination of information among relevant authorities. It was observed that lower-income women viewed contraception as expensive, likely influencing their decision on usage and limiting access (p=0.005). It was noted that women with higher education had a positive perception of contraceptive use (p=0.008). Women with higher income (p=0.003), education (p=0.005), and employment (p=0.031) also demonstrated better knowledge on contraceptive use.

The limitations of the study were that it was conducted within a hospital setting, and the findings may not be representative of the general population. Some women may have provided socially desirable answers about contraceptive use rather than their actual practice.

## Conclusions

The awareness of contraceptives among participants was good, but knowledge of the use of specific methods of contraception was poor. Study participants with higher education level, income status, and parity were associated with increased contraceptive use. A significant number of women cited fear of side effects and health concerns as reasons for discontinuation/non-use of contraceptives.

There is a need for sustained information dissemination by government, health care professionals, opinion leaders, as well as traditional and religious leaders, if the misperceptions about contraceptive use are to be dispelled and the uptake of contraceptives improved.

## Appendices

Questionnaire

Section 1

Demographic Data

Respondents No

1. Age

a. >20yrs b. 20-35yrs c. >35yrs

2. Education

a. illiterate b. primary c. secondary d. tertiary 

3. Occupation

a. housewife b. unemployed c. employed d. employer

4. Religion

a. Christian b. Muslim c. other

5. No of living children

a. None b. 1-3 c. less than 3

6. Income 

a. low income b. middle income c. high income

7. Decision maker about contraception

a. Mutual b. Husband/Wife c. In-Laws

Section 2

5-Point Likert Scale to Identify Perception on Use of Contraceptives

Introduction: Please read the following statement and rate how much you personally agree or disagree with these statements by ticking in appropriate columns.

**Table 13:** 5-point Likert scale

/N	Perception	Strongly Agree	Agree	Neither Agree Nor Disagree	Disagree	Strongly Disagree
1	Contraception are only for adult married people					
2	Contraceptives are expensive					
3	Adolescents who use contraceptives are bad					
4	Contraceptive use leads to infertility					
5	The process of acquiring contraceptive is often embarrassing					
6	I approve use of contraceptive					
7	Contraceptives are effective in avoiding pregnancy					
8	Advertisement and information about contraceptive use is immoral					
9	Contraceptives have significant side effects					
10	Religion prohibits the use of contraception					

Instructions: Respondents are requested to tick the suitable option

1. Do you use any contraceptive?

a) Yes     b)No

if yes then, please specify

**Table 14:** Contraceptive methods

Contraceptives	Tick the Appropriate	Duration of use
1	Oral Contraceptive Pills		
2	Condoms		
3	Cu-T		
4	Male Sterilization		
5	Female Sterilization		
6	Hormonal IUD		
7	Implant		
8	Injectibles		
9	Emergency contraception		
10	Natural method		

If no, then please tick the appropriate

**Table 15:** Reason for non use/discontination

S/N	Reasons For Non Usage/ Discontinuing The Contraceptive Method	Tick The Appropriate
1	Method failure	
2	Desire to become pregnant	
3	Side effects/Health concerns	
4	Costly	
5	Infrequent sex/Partner away	
6	Inconvenient to use	
7	Religious prohibition	
8	Lack of knowledge regarding method and source	
9	Switch to other method	
10	Other reasons	

Section 3

a. Condoms

1) When does your partner use condom

a) Before erection

b) After erection 

c) During erection 

2)   What technique will he use while wearing?

 a)  Rolling over erect penis

 b) Unroll it before wearing

 c)  Other technique

3) When should he remove the condom? 

 a) Before ejaculation

  b) Soon after ejaculation

b. Oral Pills

 1) How often will you take a pill?

 a) Once a month

b) Once a day 

  c) Before having sex

 d) Don’t know

 2) What will you do if you forget to take a pill?

a) Take two the next day 

 b) Stop taking the pills until she gets her period 

 c) Whenever you will remember and continue taking pills next day

3) When will you start the pill?

a) First day of menstrual period

b) Last day of menstrual period

c) Any day of the cycle 

c. IUD

1) What is the ideal time of insertion

a) During or just after the menstrual cycle

b) When you are pregnant

c) None of the above 

2) How will you determine the proper position of IUD?

a) Thread stays

b) Thread hangs outside

c) None of the above

3) How long can IUD be used? 

a) Five to ten years

b) More than ten years 

c) Lifetime

d. Injectibles

1) How frequently is the injection taken

a) Every 2-3weeks

b) Every 2-3months

c) Every 2-3 years

2) When is the ideal time to take the injection

a) Before due date

b) On due date

c) After due date

e. Tubal Ligation

1. What type of contraceptive method is this

a) Temporary

b) Permanent

2) Do you need further protection after procedure

a) Yes

b) No

c) I don’t know

f. Implant

1. They can affect your fertility in the future

a) Yes

b) No

c) I don’t know

2. How long can they be used

a) Less than 5years

b) 5 years or more

c) None of the above

g. Emergency Contraception

1. When should it be used?

a) Within 5 days of unprotected intercourse

b) Within 2 weeks of unprotected intercourse

c) At anytime

2. Emergency contraception is a form of abortion

a) Yes

b) No

c) I don’t know

3 Early use of emergency contraception can help prevent STI

a) Yes

b) No

c) I don’t know
